# Chronic Appendicitis in Its Relation to Dyspepsia

**Published:** 1917-04

**Authors:** H. Goodwyn

**Affiliations:** Bovey Tracey


### CHRONIC APPENDICITIS IN ITS RELATION TO DYSPEPSIA.

BY

H. Goodwyn, F.R.C.S. Ed.,

*Bovey Tracey.*

I do not claim any originality for my views on this subject, and it is with a view to emphasise the experience of others that I venture to record my experience. Some years ago Sir Berkeley Moynihan published a paper on this subject, and advanced the opinion that there was a very large number of cases of chronic and even acute dyspepsia that were directly dependent on an irritable condition of the appendix, whether as the result of acute or subacute attacks, or simply on some abnormal condition of the appendix and its surroundings, never sufficiently severe or definite to attract attention to that organ. Some time since Mr. Carwardine also drew attention to this condition in a paper he published in the *British Medical Journal,* [which was illustrated with drawings showing the findings at operation], and especially to the presence in a number of cases of the so-called " Jackson's membrane."

It seems to be a matter not yet definitely settled as to whether this latter is of an inflammatory nature or congenital. In the several cases I have found it present I am convinced that it is pathological, but I do not wish to be decided on the point, as I have seen too few cases. This paper emphasised the importance of the connection between the appendix condition and the chronic dyspeptic troubles most of the cases were suffering from, and which were, in all the cases submitted to operation, either cured or greatly relieved.

It would be outside the scope of my paper to review the whole vexed subject of appendicitis, the arguments for or against operation, when to operate, etc. I merely wish to place on record my small experiences, in the hope that they may encourage a more extended trial of removal of the organ where there is any evidence at all pointing to its having some abnormal condition.

We might first ask ourselves why should an irritable appendix, which is not in direct relation to the stomach, give rise to trouble in that organ. I think the answer is to be found in the intimate connection of the sympathetic nervous system, the arrangements of Auerbach's and Meisner's plexuses in the coats of the small and large intestines, and the sympathetic nerve supply to the stomach itself.

The different types of ailments which present themselves may be very varied in severity, ranging from mildly persistent indigestion to even severe gastric hemorrhage, though the commonest type in my experience is that of chronic, intractable dyspepsia. Constipation ; the constant need for aperients is a constant source of worry, until one might almost think the case was one of gradually increasing obstruction. It is seldom that the ordinary treatment by drugs and diet is of much avail, and then they are only palliative. This brings us to the fact that all cases of intractable dyspepsia should have the appendix region carefully explored, both by abdominal and rectal examination, and that an X-ray examination with a bismuth meal should never be omitted, where possible, by an expert accustomed to this class of work. It is unnecessary to say that one would first endeavour to exclude all other possible causes of a more serious nature, such as gastric ulcer, duodenal ulcer, carcinoma of the stomach or other neighbouring organ, etc., etc., but the class of case to which I am alluding seldom or never presents serious symptoms. My experience extends over a period of six years, during which I have operated on a fair number of cases, all of whom have been cured of their dyspepsia.

It is unnecessary to quote all the cases, but the few following are sufficient to illustrate the points to which I desire to direct attention :—

1. Miss L. complained of attacks of vomiting and acute pain in the stomach, coming on at intervals of a fortnight or three weeks, and rendering her quite unfit for anything. She had marked tenderness on deep pressure over the appendix, no history of an acute attack, but these attacks had gone on for twenty years. At operation the appendix was found buried in the wall of the cœcum by membranous tough adhesions, and really at first one was inclined to think there was no appendix at all, it was so difficult to find. It was removed, and the recovery was uneventful. I asked her to write me six months after, and she told me she was quite free from all her former troubles, could eat anything, and had not felt so well for twenty years.

2. A. W. had frequently suffered from attacks of acute indigestion, lasting several weeks at a time. Medicine and diet only gave temporary relief. I was called to see him one night, and found him in great pain, vomiting, some distension, pulse 100, temperature 100 ; vomiting had been going on for some hours, and it was evidently either an acute appendix or acute obstruction. I operated the next morning, and found the appendix firmly adherent at the tip to the abdominal wall up behind the cœcum, and a knuckle of intestine was being slowly obstructed, and the colour was dark purple. It was very difficult to free the tip, and the proximal end had to be detached from the bowel before the tip could be cleared. The recovery was uneventful, and now, a year after, he is perfectly well, free from all indigestion, and has gained two stone in weight.

3. Mrs. R. had suffered from indigestion all her life. Had all sorts of treatment—diet, teeth all removed and new set fitted; very constipated, and loathed her food; could only eat the simplest things, often had to go on a milk diet for weeks at a time. At operation a long, irritable appendix was found, and the greater part was enveloped in a membranous fold spreading over the cœcum (Jackson's membrane). This was separated and the organ removed. She is now quite well, and has put on over a stone in weight, and is able to eat anything, even roast pork, with impunity!

4. A. W. This case is of interest on account of the finding of a bristle in the appendix. The patient had been out of health for months, attacks of indigestion of varying severity, and loss of flesh. A point of deep tenderness over the appendix. Complete recovery from all symptoms after removal.

5. W. G. This was not a patient of mine, but was operated on in London by my advice for the same train of troubles common to most. He had attacks of acute vomiting, very poor digestion at any time, and was very thin. A bristle was found in his case also, and he is now a different man.

6. B. I. Complained of the usual chronic pain after food, no treatment had done her any real good, and on examination a point of tenderness was present. Appendix was very long and twisted, with bulbous end, small concretions in the lumen, and signs of old ulceration of the mucous lining. Removal caused all symptoms to disappear, and she is now in perfect health.

It would be tedious to quote further cases, and I hope those mentioned will be sufficient to show the value of operation in suitable cases. In conclusion, I would emphasise again the value of a careful examination of all the appendix region where intractable indigestion is a prominent symptom.

